# Case Report: Anti-NMDA Receptor Encephalitis With Bilateral Hearing Loss as the Initial Symptom

**DOI:** 10.3389/fneur.2021.648911

**Published:** 2021-03-17

**Authors:** Hongjiang Cheng, Fengbing Yang, Jing Zhang, Lina Xu, Longbin Jia, Doudou Zhao, Wei Liu, Huimin Li

**Affiliations:** ^1^Jincheng People's Hospital Affiliated to Shanxi Medical University, Jincheng, China; ^2^Changzhi Medical College, Changzhi, China

**Keywords:** autoimmune disease, autoimmune encephalitis, anti-NMDAR encephalitis, hearing loss, case report

## Abstract

**Introduction:** Anti-*N*-methyl-D-aspartate receptor (anti-NMDAR) encephalitis is an autoimmune disease associated with the NMDA receptor. This paper describes a patient who presented with bilateral hearing loss as the initial symptom of anti-NMDAR encephalitis.

**Case Report:** We describe a 31-year-old young female with anti-NMDAR encephalitis who presented with bilateral severe hearing loss after brief loss of consciousness and then accompanied by other symptoms, such as generalized tonic–clonic seizures, manic episodes, excessive salivation, severe cognitive impairment, and complex non-convulsive status epilepticus. Great improvement was achieved by a combined treatment of steroid, IVIG, and plasmapheresis, especially after surgical removal of the ovarian teratoma. When she was discharged on hospital day 43, her hearing loss obtained a significant improvement.

**Conclusion:** This case study and literature review investigated the impairment of hearing loss and its subsequent treatment in patients with anti-NMDAR encephalitis.

## Introduction

Anti-*N*-methyl-d-aspartate receptor (anti-NMDAR) encephalitis is an autoimmune disease associated with IgG antibodies against the NR1 subunit of the NMDA receptor (1). Patients usually present with acute behavioral change, psychosis, and catatonia that evolve to include seizures, memory deficit, dyskinesias, speech problems, and autonomic and breathing dysregulation (2), but hearing loss is rare. Here, we report a patient who presented with bilateral serious hearing loss as the initial symptom of anti-NMDAR encephalitis.

## Case Report

**Figure 1:**
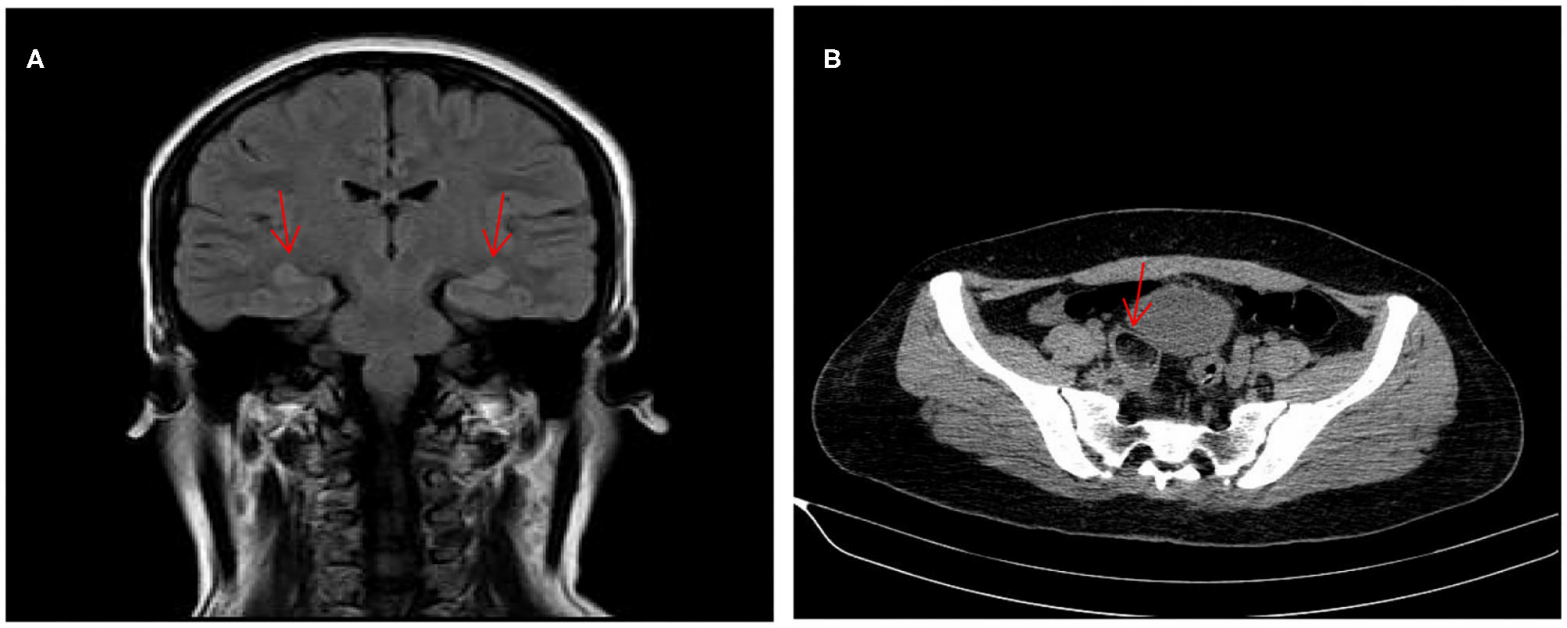
MRI of the brain revealed subtle increased signal intensity in bilateral hippocampal on FLAIR sequences **(A)**. CT scan of the abdomen indicated a space-occupying lesion in right-side ovarian, later pathologically identified as mature ovarian cystic teratoma **(B)**.

**Figure 2:**
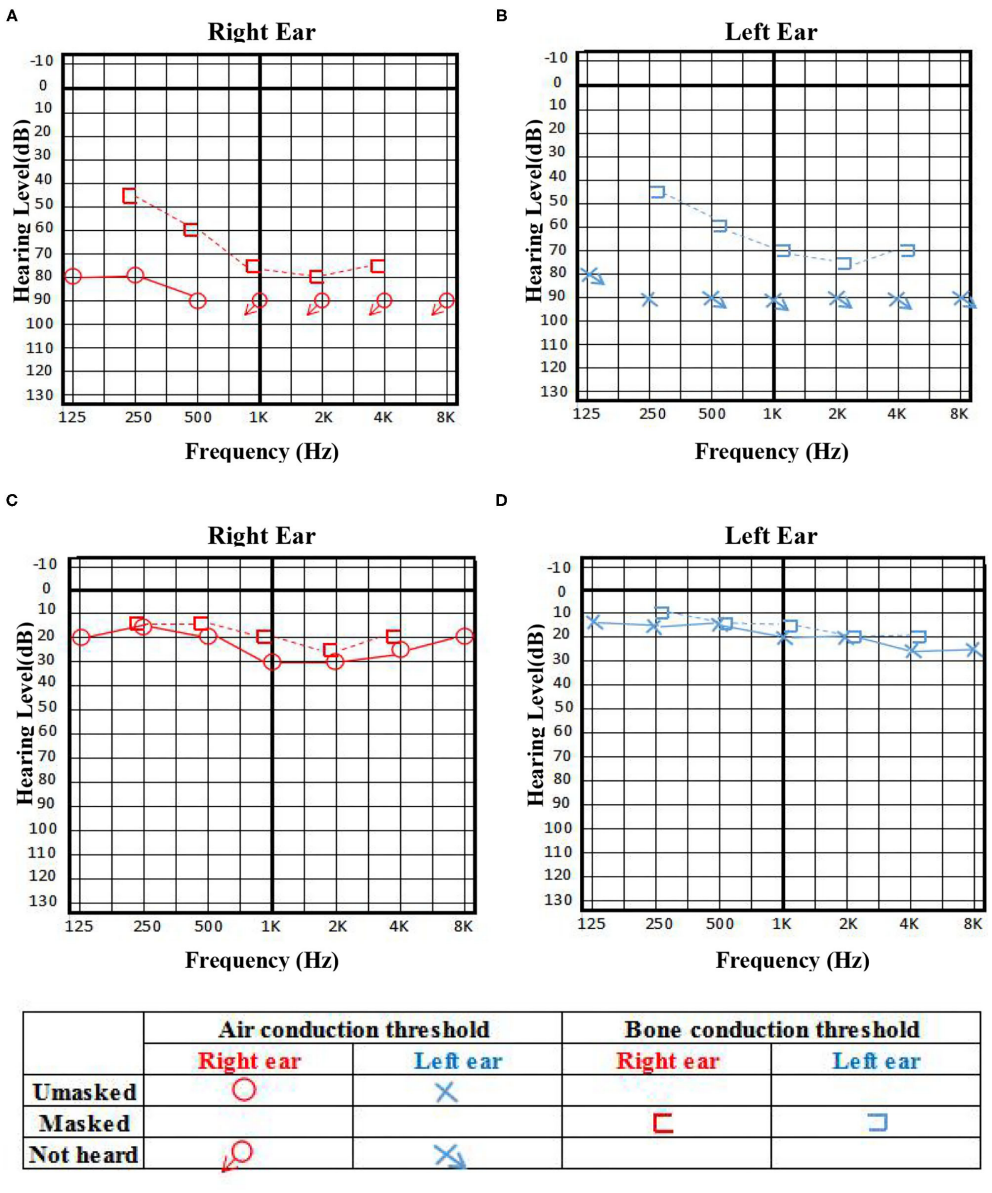
The pure tone audiogram on admission indicated bilateral serious sensorineural hearing loss, predominantly in higher frequencies **(A,B)**, and achieved significant improvement 3 days before discharge **(C,D)**.

A 31-year-old young female with no previous underlying disease presented to the Otolaryngology Department complaining of hearing loss in both ears after brief loss of consciousness, without tinnitus and vertigo. Three days prior to presentation, she had an intermittent cough. Head MRI examination detected subtle increased signal intensity in bilateral hippocampal on FLAIR sequences (Figure 1A). The otoscopic examination showed no evidence of the external auditory canal and tympanic membrane abnormality, yet pure tone audiogram indicated bilateral serious sensorineural hearing loss, predominantly in higher frequencies (Figure 2). Then, she was transferred to the Department of Neurology.

On admission, a physical examination showed bilateral hearing loss and positive Kerning's sign without other neurologic signs or symptoms. In the evening, she had a 5-min tonic–clonic seizure. Then, she woke up, finding that her hearing had restored dramatically. However, the hearing was lost again the next morning. Two-hour electroencephalogram revealed continuous low-amplitude β activity in the bilateral occipital region, mixed with a little more 10–12 Hz low-amplitude α activity, which was approximately symmetrical, and the occipital rhythm could be inhibited when the eyes were opened. She was initiated on sodium valproate 400 mg three times daily. A lumbar puncture was performed to this patient on the second day of admission. The cerebrospinal fluid (CSF) showed an increased white blood cell (WBC) count (60 × 10^6^/L, reference range 0–8 × 10^6^/L) with a lymphocytic predominance and normal protein and glucose levels. On hospital day 3, the symptoms of this patient were progressing rapidly, developing manic episode, language impairment, and increased salivation. Her manic symptom was gradually controlled when treated with midazolam or diazepam intravenous infusion and risperidone tablets (1 mg twice daily). Since our patient had such signs, the diagnosis of autoimmune encephalitis was suspected. A 5-day course of IV methylprednisolone (1 g/day) and intravenous immunoglobulin (IVIG) (25 g/day) were administered empirically. On hospital day 4, the patient showed significant cognitive dysfunction and even did not know where she came from. CSF PCR for herpes simplex virus (HSV) 1 and 2 was unremarkable. Serum and CSF anti-NMDAR antibodies returned positive by immunofluorescence (1:10 serum, 1:100 CSF), confirming the diagnosis of definite anti-NMDAR encephalitis (3). On hospital day 8, the signs of saliva leakage were gone and hearing in the left ear showed some extent of retrieval, although it was not sustained. Occasionally, she could hear us when we shouted. However, her speech-language disorder became more and more serious, and she even could not express a word completely, displaced by aimless smirk. Her psychiatric symptoms were serious and she shouted sometimes. Quetiapine (the starting dose was 50 mg once daily) was added to control her significant manic symptoms.

**Figure 3:**
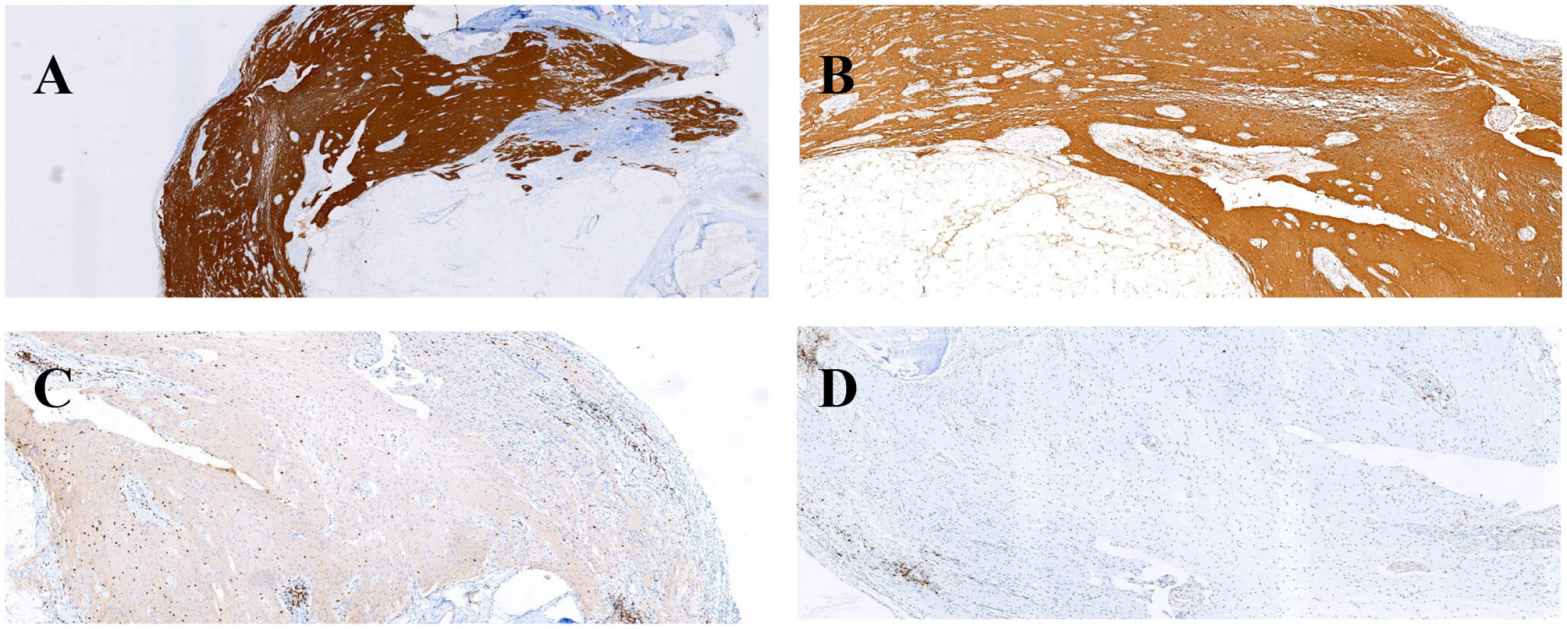
Immunohistochemical findings **(A,B)** and inflammatory response **(C,D)** in this patient's ovarian teratoma. GFAP **(A)** and S-100 **(B)** immunohistochemical staining were positive. Infiltration of scattered CD3 T-cells **(C)** and CD20 **(D)** B-cells.

With the suspicion of ovarian teratoma in our patient, abdominal CT was performed on hospital day 10, which indicated a space-occupying lesion (2.0 × 3.0 cm) in the right-side ovary (Figure 1B). Because of the poor condition of the patient, two sessions of plasmapheresis were done within 3 days. On hospital day 14, the patient underwent laparoscopic ovarian cystectomy, and a pathological examination revealed a mature ovarian cystic teratoma, containing nervous tissue (Figure 3). Three other plasmapheresis sessions were conducted in the following 8 days. During this period, her neurologic condition progressively improved. Her hearing recovered significantly. She could make some simple actions according to our directions, such as raising her hand or opening her mouth. Symptoms, such as mental irritability, were almost non-existent. We gradually discontinued antipsychotic medication, including risperidone, quetiapine, and midazolam. The language ability also improved to some degree. She could tell us her home address accurately. On hospital day 23, our patient presented involuntary muscle contractions on both sides of the face. The next day, she developed consciousness disturbances and uninterrupted and involuntary mastication without convulsions. When diazepam (10 mg) was intravenously injected, the patient woke up immediately and could answer our questions. In the following hours, there were still several similar episodes and diazepam was effective. The diagnosis of non-convulsive status epilepticus was considered. Midazolam was given intravenously at a sustained dose of 2 mg/h. Under the treatment, epilepsy was well-controlled. It was possible that due to the use of sedation medications, epileptiform discharges could not be found on subsequent EEG, showing 8–9 Hz low-amplitude α rhythm in the bilateral occipital region, mixed with a small amount of low-amplitude fast wave activity, which was approximately symmetrical, and the occipital rhythm could be inhibited when the eyes were opened. Two weeks later, the cognitive tests were administered to our patient. The MMSE score was 18 and the MoCA score was 14. The patient still showed some degree of cognitive dysfunction, such as comprehension, memory, calculation, etc. Fortunately, her hearing loss obtained a significant improvement. Pure tone audiogram indicated nearly normal hearing in both ears (Figure 2). A lumbar puncture was performed once more on the day before our patient discharge. The results suggested that anti-NMDAR antibodies were still positive (1:30 serum, 1:100 CSF). The patient was discharged on hospital day 43. She could perform activities of daily living independently.

**Figure 4:**
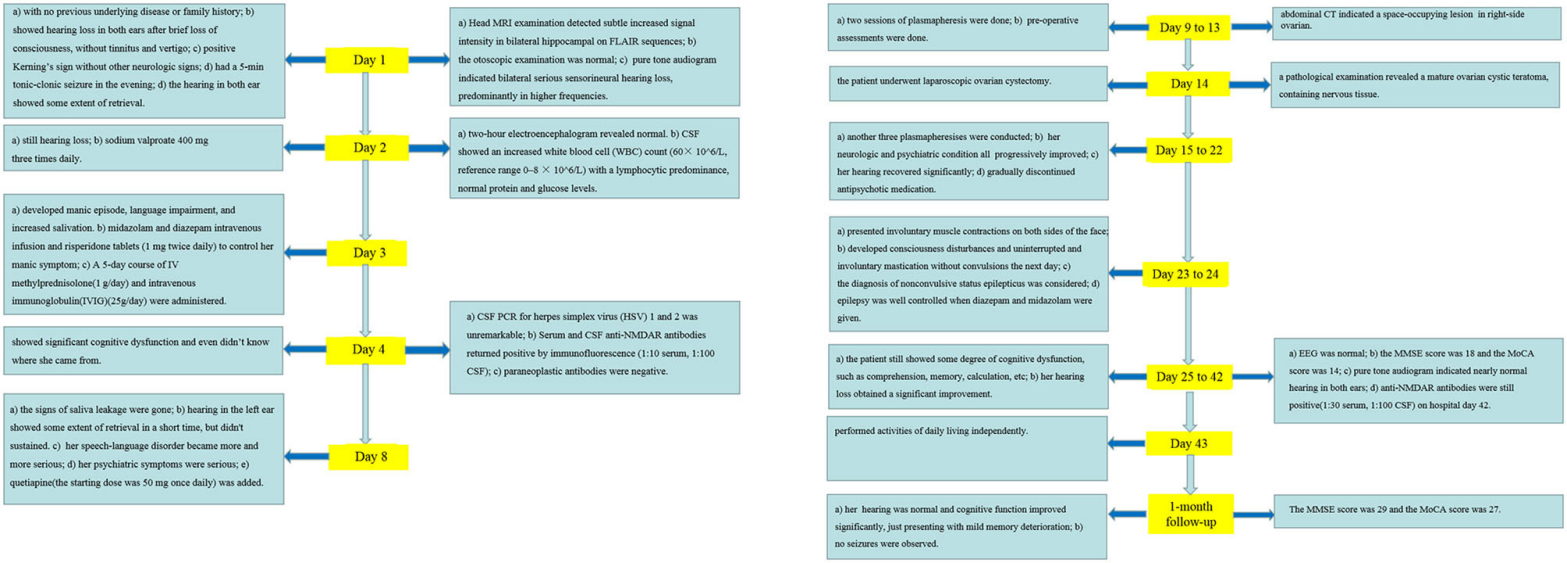
Clinical course of the case.

In the 1-month follow-up, her hearing was normal and cognitive function improved significantly, just presenting with mild memory deterioration. During this period, no seizures were observed. The MMSE score was 29 and the MoCA score was 27. Regular follow-up will be carried out for our patient. The clinical course is summarized in Figure 4.

## Discussion

Anti-NMDAR encephalitis was first identified in 2007 by Dalmau et al. (1). The clinical manifestations of patients with this disease are complex and vary in severity. About 70% of patients have prodomal symptoms consisting of headache, fever, nausea, vomiting, diarrhea, or upper respiratory tract symptoms. Within a few days, usually <2 weeks, patients develop a multistage illness that progresses from psychosis, memory deficits, seizures, and language disintegration into a state of unresponsiveness with catatonic features often associated with abnormal movements, and autonomic and breathing instability (4). However, it is rare that the patient present with bilateral hearing loss of sudden onset and few reports are available. The pathogenesis of bilateral fluctuating hearing loss is unknown and the diagnosis is difficult. Ictal deafness is a focal auditory seizure characterized by suppression of hearing, presumably originating from the auditory cortex in the temporal lobe (5). Only 12 cases have been reported. Patients generally present with an inability to hear during a seizure and are normal in the interictal period. The left temporal area is usually the most common site of abnormal discharges and the right temporal area is rare (5–8). The seizures recur repeatedly and last several minutes or more. When antiepileptic medication is given, most patients achieve a good therapeutic effect. Our patient presented with bilateral serious sensorineuronal hearing loss as the initial symptom and then developed generalized tonic–clonic seizures and non-convulsive status epilepticus. Multiple EEG examinations were completed, and no similar epileptiform activity was seen in our patient. Although anticonvulsant drugs such as midazolam or diazepam were administered repeatedly, the patient's hearing recovery seemed to be inconspicuous. Additionally, her hearing loss lasted longer and she failed to obtain significant recovery even in the interictal period. It seemed that sometimes she could hear a few sounds in the left ear when we shouted, but the hearing only lasted for a short time. This is contrary to what has been reported in the literature (5–8).

Paraneoplastic syndrome has also been reported in some literature associated with hearing loss. The most frequent is Kelch-like (KLHL) 11 encephalitis that is associated with testicular germ cell tumors, benign teratomas, small-cell lung cancer, and ovarian cancer (9). The typical clinical presentation is rhomboencephalitis, with predominant ataxia, diplopia, dysarthria, hearing loss, tinnitus, and vertigo. Hearing loss and tinnitus appear to be relatively unique manifestations of KLHL11 encephalitis, which preceded other neurologic deficits for months (10). Most cases occur in men and a few are women (9–11). In a recent article from Barcelona, the authors found that some patients with well-defined syndromes (anti-NMDAR encephalitis) had concurrent Kelch-like protein 11 (KLHL11-ab) (9). The hearing loss is difficult to recover, and only 25% of cases are noted to improve neurologically following cancer treatment and/or immunotherapy (10). The anti-Hu antibody is the second most frequent antibody associated with hearing loss, as reported in some literature (12–16). The patients in some cases with anti-Hu antibody presented as various degrees of sensorineural hearing loss monaural or binaural. Tumors, such as small cell lung carcinoma (SCLC), neuroblastoma, and so on, are ultimately confirmed in most patients through subsequent detailed examination. Encephalitis associated with anti-Hu antibodies is usually not improved by immunosuppression, plasmapheresis, or IVIG (17). The early therapeutic intervention of the underlying tumor before neurologic deterioration and death occurs may be effective. In addition, some other antibodies such as anti-AChR Ab, anti-VGKC Ab, anti-GAD65 Ab, anti-Yo, anti-Ri, anti-Tr, anti-CV2/CRMP5 (Collapsin response mediator protein 5), anti-Ma, and anti-amphiphysin also have a close relationship with paraneoplastic cochleovestibulopathy (18–22). Paraneoplastic cochleovestibulopathy is a rare manifestation of paraneoplastic neuronal disorders involving sudden onset bilateral sensorineuronal hearing loss refractory to high-dose oral, IV, or intratympanic steroids, with worsening neurologic symptoms such as ataxia, vertigo, and opsoclonus-myoclonic seizures and could be confirmed by either a known paraneoplastic antibody or evidence of an occult neoplasm, often leading to death (13–15, 23). Our patient was a young woman presented with bilateral serious sensorineural hearing loss, and no tumors were detected at a subsequent examination. Paraneoplastic antibodies (anti-Hu, anti-Yo, anti-Ri, anti-CV2, anti-amphiphysin, anti-Ma2-Ta, anti-Ma1, anti-SOX1, anti-Zic4, anti-GAD65, and anti-DNER) were negative, and her hearing improved with the combined treatment of steroid, IVIG, and plasmapheresis, especially after surgical removal of ovarian teratoma. Unfortunately, KLHL11-abs were not examined in our patient, and we are not sure if this patient had concurrent KLHL11-abs.

The strongest pathogenic evidence of bilateral fluctuating hearing loss is that of an immune-mediated disease. The inner ear is not an immunologically privileged site and may mount an immune response against foreign and self-antigens damaging sensory structures within it (24–26). Both arms of the immune system, innate and adaptive, are implicated in the processes ultimately leading to the histopathological features in the cochlea of patients with autoimmune inner ear disease (27–29). One suggested pathway is through upregulation of interleukin-1b in the fibrocytes in the spiral ligament, priming the inner ear to allow a small number of leucocytes to enter. Subsequently, in individuals with lymphocytes primed to react against inner-ear antigens, an immune response may be initiated (30). Xin et al. (31) argued that NMDARs were expressed in SGN dendritic terminals and played a critical role during the transmission of activity from IHCs to SGNs before hearing onset. It is possible that NMDA receptors mediated in the inner ear may be involved in hearing impairment in this patient. Autoimmune hearing loss is also observed from Wegener's granulomatosis, Cogan's syndrome, rheumatoid arthritis, systemic lupus erythematosus, polyarteritis nodosa, or relapsing polychondritis (32, 33). The patient had no history of rheumatic diseases, and we did not observe rheumatoid and immune-related abnormalities, such as rheumatoid factor, ANA, anti-CCP, ANCA, etc. Therefore, we basically denied these diagnoses.

In this case, the patient presented with bilateral severe hearing loss when she visited our hospital. Serum and CSF anti-NMDAR antibodies were positive, confirming the diagnosis of definite anti-NMDAR encephalitis. During the treatment with methylprednisolone, gamma globulin, and plasmapheresis intravenously, the hearing showed a degree of volatility. Occasionally, sound could be heard in the left ear, but it did not sustain. Starting from the second day after the surgical excision of ovarian teratomas, binaural hearing of this patient showed some extent of retrieval, especially after the following three sessions of plasmapheresis. Fortunately, the hearing loss in this young patient recovered substantially after 43 days of treatment. So far, the hearing loss in anti-NMDA receptor encephalitis associated with ovarian teratoma has not been reported. Taraschenko et al. (34) reported that a male with anti-NMDAR encephalitis showed atrial fibrillation and sensorineural hearing loss. Despite the patient receiving treatment with corticosteroids, IVIG, and rituximab, the therapeutic effect was unsatisfactory. The treatment approach to anti-NMDAR encephalitis involves escalation of immunotherapy, starting with first-line therapies (steroids, IVIG, or plasma exchange) and transitioning to second-line therapies (rituximab or cyclophosphamide) if needed (35, 36). Dalmau argued that resection of the tumor associated with anti-NMDAR encephalitis appeared to be important to attain final recovery or sustain the improvement that in some cases started soon after immunotherapy (corticosteroids, IVIG, or plasma exchange) (1). Hearing loss related to anti-NMDAR encephalitis, as one kind of autoimmune inner ear disease, may benefit from the treatment of steroid, cytotoxic agents, immunosuppressive drugs, and biologic agents (37–40).

## Conclusion

In summary, our case, as the first episode of anti-NMDAR encephalitis, initially presented with bilateral hearing loss after brief loss of consciousness and then accompanied by other symptoms, such as generalized tonic–clonic seizures, manic episodes, excessive salivation, severe cognitive impairment, and complex non-convulsive status epilepticus. Great improvement was achieved by a combined treatment of steroid, IVIG, and plasmapheresis, especially after surgical removal of ovarian teratoma. An awareness of diverse and uncommon primary manifestations is conducive to early and accurate diagnosis of anti-NMDAR encephalitis.

## Data Availability Statement

The original contributions presented in the study are included in the article/Supplementary Material, further inquiries can be directed to the corresponding authors.

## Ethics Statement

The studies involving human participants were reviewed and approved by Jincheng people's Hospital, Jincheng, China. The patients/participants provided their written informed consent to participate in this study.

## Author Contributions

LJ: put forward research ideas. FY: took the responsibility of communicating with the patient's family and obtaining the authorization in this paper. HC and JZ: responsible for drafting articles. All authors contributed to the article and approved the submitted version.

## Conflict of Interest

The authors declare that the research was conducted in the absence of any commercial or financial relationships that could be construed as a potential conflict of interest.
